# Comparing the safety and efficacy of ruxolitinib in patients with Dynamic International Prognostic Scoring System low‐, intermediate‐1‐, intermediate‐2‐, and high‐risk myelofibrosis in JUMP, a Phase 3b, expanded‐access study

**DOI:** 10.1002/hon.2898

**Published:** 2021-07-05

**Authors:** Francesco Passamonti, Vikas Gupta, Bruno Martino, Lynda Foltz, Andrey Zaritskey, Haifa Kathrin Al‐Ali, Renato Tavares, Margherita Maffioli, Pia Raanani, Pilar Giraldo, Martin Griesshammer, Paola Guglielmelli, Catherine Bouard, Carole Paley, Ranjan Tiwari, Alessandro M. Vannucchi

**Affiliations:** ^1^ University of Insubria Varese Italy; ^2^ Princess Margaret Cancer Centre Toronto Ontario Canada; ^3^ Azienda Ospedaliera Bianchi‐Melacrino‐Morelli Reggio Calabria Italy; ^4^ St Paul's Hospital University of British Columbia Vancouver BC Canada; ^5^ Federal Almazov Medical Research Center of the Russian Ministry of Health St Petersburg Russia; ^6^ University Hospital of Halle Halle (Saale) Germany; ^7^ Universidade Federal de Goiania Goiânia Brazil; ^8^ Ospedale di Circolo, ASST Sette Laghi Varese Italy; ^9^ Rabin Medical Center Petah Tikva and Sackler School of Medicine Tel Aviv University Tel Aviv Israel; ^10^ Miguel Servet University Hospital and Centro de Investigacion Biomedica en Red de Enfermedades Raras (CIBERER) Zaragoza Spain; ^11^ Johannes Wesling Clinic, University of Bochum Minden Germany; ^12^ CRIMM Center for Research and Innovation of Myeloproliferative Neoplasms AOU Careggi University of Florence Florence Italy; ^13^ Novartis Pharma Rueil‐Malmaison France; ^14^ Novartis Pharma East Hanover New Jersey USA; ^15^ Novartis Healthcare Pvt., Ltd. Hyderabad Telangana India

**Keywords:** Dynamic International Prognostic Scoring System, efficacy, ruxolitinib, safety

## Abstract

Ruxolitinib, a potent Janus kinase 1/2 inhibitor, has demonstrated durable improvements in patients with myelofibrosis. In this analysis of the Phase 3b JUMP study, which included patients aged ≥18 years with a diagnosis of primary or secondary myelofibrosis, we assessed the safety and efficacy of ruxolitinib in patients stratified by Dynamic International Prognostic Scoring System (DIPSS) risk categories. Baseline characteristic data were available to assess DIPSS status for 1844 of the 2233 enrolled patients; 60, 835, 755, and 194 in the low‐, intermediate (Int)‐1‐, Int‐2‐, and high‐risk groups, respectively. Ruxolitinib was generally well tolerated across all risk groups, with an adverse‐event (AE) profile consistent with previous reports. The most common hematologic AEs were thrombocytopenia and anemia, with highest rates of Grade ≥3 events in high‐risk patients. Approximately, 73% of patients experienced ≥50% reductions in palpable spleen length at any point in the ≤24‐month treatment period, with highest rates in lower‐risk categories (low, 82.1%; Int‐1, 79.3%; Int‐2, 67.1%; high risk, 61.6%). Median time to spleen length reduction was 5.1 weeks and was shortest in lower‐risk patients. Across measures, 40%–57% of patients showed clinically meaningful symptom improvements, which were observed from 4 weeks after treatment initiation and maintained throughout the study. Overall survival (OS) was 92% at Week 72 and 75% at Week 240 (4.6 years). Median OS was longer for Int‐2‐risk than high‐risk patients (253.6 vs. 147.3 weeks), but not evaluable in low‐/Int‐1‐risk patients. By Week 240, progression‐free survival (PFS) and leukemia‐free survival (LFS) rates were higher in lower‐risk patients (PFS: low, 90%; Int‐1, 82%; Int‐2, 46%; high risk, 15%; LFS: low, 92%; Int‐1, 86%; Int‐2, 58%; high risk, 19%). Clinical benefit was seen across risk groups, with more rapid improvements in lower risk patients. Overall, this analysis indicates that ruxolitinib benefits lower‐risk DIPSS patients in addition to higher risk.

## 1 INTRODUCTION

Ruxolitinib is a potent Janus kinase (JAK) 1/JAK2 inhibitor that has demonstrated durable improvements in splenomegaly, myelofibrosis (MF)‐related symptoms, and quality‐of‐life measures in patients with International Prognostic Scoring System (IPSS)^1^ intermediate (Int)‐2‐ and high‐risk MF in the Phase 3 COMFORT studies.^2^, ^3^ In COMFORT I and II, 42% and 28% of patients in the ruxolitinib groups, respectively, achieved a reduction in spleen volume of 35% or more at 24 weeks, compared with 0.7% and 0% in the placebo groups, respectively.^2^, ^3^ In post hoc analyses of the COMFORT studies, ruxolitinib was associated with improved survival rates versus placebo^2^ and best available therapy^3^, ^4^ and also when compared with historical matched cohorts.^5^

In primary MF, the IPSS is used for initial diagnosis of patients.^1^ In this system, increasing numbers of five independent predictors of inferior survival (age >65 years, hemoglobin <10 g/dl, leukocyte count >25 ×10^9^/L, circulating blasts more than 1%, and the presence of constitutional symptoms) define risk categories of low‐, Int‐1‐, Int‐2‐, and high‐risk disease. Subsequently, the Dynamic International Prognostic Scoring System (DIPSS) model was developed and utilizes the same prognostic variables as in the IPSS, but with differences in scoring (e.g., DIPSS assigns 2 rather than 1 point for hemoglobin <10 g/dl).^6^ The DIPSS can be applied at any time during the disease course and includes risk categories of low (0 adverse points), Int‐1 (1‐2 points), Int‐2 (3–4 points), and high (5–6 points), with corresponding median survival rates of not reached, 14.2, 4.0, and 1.5 years, respectively. As such, the DIPSS risk categories reflect the dynamic nature of the enrollment period of patients into a trial in a manner that the IPSS categories cannot.

JAK Inhibitor RUxolitinib in Myelofibrosis Patients (JUMP) is a large, single‐arm, open‐label, Phase 3b trial, including IPSS Int‐1‐, Int‐2‐, and high‐risk MF patients (Clinicaltrials.gov identifier: NCT01493414).^7^ In the current analysis, we explored the safety and efficacy of ruxolitinib in patients in the JUMP study who were stratified according to the DIPSS. As only the IPSS was available when the JUMP and COMFORT study protocols were developed, this subgroup analysis was performed to give insight into any safety or efficacy differences of ruxolitinib across DIPSS risk categories and to allow comparisons with future studies using these criteria. By considering this time‐dependent risk classification (DIPSS) that more accurately reflects the dynamic enrollment period of the JUMP trial, this post hoc analysis allows the impact of disease‐risk group on outcomes in the JUMP trial to be appreciated in a manner that more closely reflects clinical practice, where patients can receive treatment both at the time of diagnosis and afterward.

## 2 METHODS AND PATIENTS

Full details of the study methodology have been published previously.^7^, ^8^ In brief, patients aged ≥18 years with a diagnosis of primary or secondary MF by World Health Organization and International Working Group for Myeloproliferative Neoplasms Research and Treatment (IWG‐MRT) criteria^9^, ^10^ and classified by the treating investigator as high, Int‐2, or Int‐1 risk using IPSS criteria, were eligible for enrollment. Patients were stratified by DIPSS based on baseline patient characteristics into low‐, Int‐1‐, Int‐2‐, and high‐risk categories. Further details of inclusion criteria have also been published previously.^7^, ^9^ Patients were treated with ruxolitinib for up to 24 months after the last patient's first visit, or until the drug became commercially available, with doses titrated from starting doses based on baseline platelet counts, from 5 mg to a maximum dose of 25 mg twice daily (b.i.d.), until discontinuation criteria were met (i.e., disease progression, unacceptable toxicity, death, discontinuation from the study for any other reason, physician decision, withdrawal of informed consent) or completed treatment per protocol, whichever occurred first (for further details, see Al‐Ali et al.^8^). The median duration of exposure to ruxolitinib was 12.4 months (range, <0.1–59.7 months).^7^ The study was conducted in accordance with the Declaration of Helsinki and principles of Good Clinical Practice. Written informed consent was obtained from all participants prior to enrollment, and the protocol and its amendments were approved by the institutional review boards of the respective institutions prior to study commencement.

The endpoints assessed in the current analysis were: proportion of patients with ≥50% reduction in palpable spleen length at postbaseline visits; patient‐reported outcomes (Functional Assessment of Cancer Therapy‐Lymphoma Total Score [FACT‐Lym TS] and Functional Assessment of Chronic Illness Therapy [FACIT] Fatigue Scale); progression‐free survival (PFS); survival without transformation to acute myeloid leukemia (leukemia‐free survival [LFS]); overall survival (OS); and safety and tolerability (rates of adverse events [AEs] and serious AEs, hemoglobin levels, and platelet counts). Response on the FACT‐Lym TS was defined as the minimally important difference (i.e., an 11.2‐point improvement from baseline).^11^ On the FACIT‐Fatigue scale, response was defined as the minimally important difference of a three‐point improvement from baseline.^12^ Given the post hoc nature of the analysis, no formal comparisons were made between the risk groups; therefore, summary statistics alone are reported. Rate of PFS was defined as the time from first ruxolitinib administration to the date of documented progression by the IWG‐MRT^13^ or death. Rates of PFS, LFS (defined as survival without transformation to acute myeloid leukemia‐free survival), and OS (defined as time from treatment initiation to death from any cause) were estimated using the Kaplan–Meier method.

## 3 RESULTS

Baseline patient disease characteristics to determine DIPSS risk status were available in 1844 of 2233 enrolled patients (low, *n* = 60; Int‐1, *n* = 835; Int‐2, *n* = 755; and high, *n* = 194; Table 1). Sixty low‐risk patients were enrolled in violation of the protocol, likely due to differences between investigator‐assessed risk and calculated risk or timing of assessment for inclusion (screening vs. baseline). These patients have been included in the analysis. IPSS risk statuses at baseline were low, *n* = 1; Int‐1, *n* = 288; Int‐2, *n* = 355; high, *n* = 255; and missing, *n* = 945. Patients with higher‐risk MF (Int‐2/high risk) according to DIPSS were older, had lower hemoglobin levels, and had higher circulating blast counts (Table 1).

**TABLE 1:** Patient characteristics at baseline

	DIPSS risk category			
	Low (*n* = 60)	Int‐1 (*n* = 835)	Int‐2 (*n* = 755)	High (*n* = 194)
Age, median (range), years	55.0 (29.0–65.0)	62.7 (18.0–88.0)	67.6 (34.0–89.0)	71.8 (43.0–88.0)
≥65 years, *n* (%)	5 (8.3)	400 (47.9)	504 (66.8)	179 (92.3)
Male, *n* (%)	38 (63.3)	460 (55.1)	401 (53.1)	105 (54.1)
Time since initial diagnosis, mean (SD), months	68.6 (81.0)	48.7 (61.2)	50.7 (63.1)^a^	54.3 (69.6)
MF subtype, *n* (%)				
PMF	32 (53.3)	478 (57.2)	473 (62.6)	121 (62.4)
PPV‐MF	17 (28.3)	229 (27.4)	152 (20.1)	34 (17.5)
PET‐MF	11 (18.3)	128 (15.3)	130 (17.2)	38 (19.6)
Missing	0	0	0	1 (0.5)
Hemoglobin level, mean (SD), g/L	127.4 (19.3)	123.0 (18.8)	98.9 (19.7)	87.0 (9.2)
<100 g/L, *n* (%)	0	23 (2.8)	485 (64.2)	194 (100.0)
Platelet count, mean (SD), ×10^9^/L	323.2 (188.4)	336.4 (220.1)	301.6 (228.7)	294.4 (253.1)
<100 × 10^9^/L, *n* (%)	0	33 (4.0)	60 (7.9)	12 (6.2)
100 to <200 × 10^9^/L, *n* (%)	16 (26.7)	221 (26.5)	259 (34.3)	71 (36.6)
≥200 × 10^9^/L, *n* (%)	44 (73.3)	581 (69.6)	436 (57.7)	111 (57.2)
Prior transfusions, *n* (%)	3 (5.0)	85 (10.2)	279 (37.0)	110 (56.7)
Peripheral blasts ≥1%, *n* (%)	0	163 (19.5)	329 (43.6)	164 (84.5)
Palpable spleen length, *n*	57	818	742	185
mean (SD), cm	13.4 (7.4)	12.2 (6.9)	13.1 (7.4)	14.5 (7.0)
Nonpalpable spleen, *n* (%)	1 (1.7)	39 (4.7)	36 (4.8)	5 (2.6)
FACT‐Lym Total Score, *n*	59	821	39	186
mean (SD)	134.8 (17.6)	115.3 (23.6)	112.8 (24.2)	108.6 (23.0)
FACIT‐Fatigue Scale, *n*	60	817	745	192
mean (SD)	41.4 (8.4)	34.2 (11.4)	31.7 (11.9)	28.1 (11.9)

Abbreviations: DIPSS, Dynamic International Prognostic Scoring System; FACIT, Functional Assessment of Chronic Illness Therapy; FACT‐Lym, Functional Assessment of Cancer Therapy‐Lymphoma; Int, intermediate; MF, myelofibrosis; PET‐MF, postessential thrombocythemia myelofibrosis; PMF, primary myelofibrosis; PPV‐MF, postpolycythemia vera myelofibrosis; *SD*, standard deviation.

^a^ *n* = 752.

**FIGURE 1:**
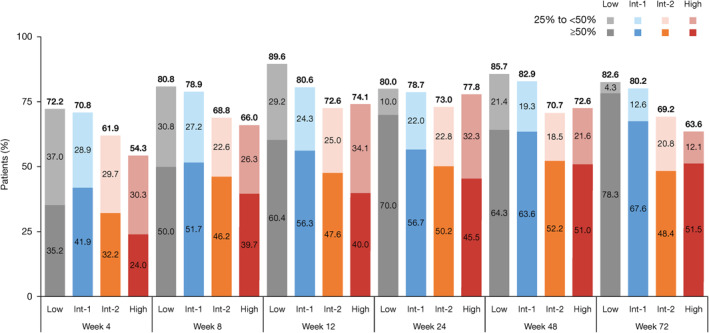
Patients with ≥25% and ≥50% reductions from baseline in palpable spleen length. Percentages of patients in each DIPSS risk group who achieved ≥25% and ≥50% reduction in palpable spleen length from baseline at time points up to Week 72 of the study. DIPSS, Dynamic International Prognostic Scoring System, Int, intermediate

Most patients (72.6%) experienced ≥50% reduction from baseline in palpable spleen length at any postbaseline visit; the proportion was highest in low‐risk (82.1%) and Int‐1‐risk (79.3%) patients compared with Int‐2‐risk (67.1%) and high‐risk (61.6%) patients. By Week 72, 78.3%, 67.6%, 48.4%, and 51.5% of low‐, Int‐1‐, Int‐2‐, and high‐risk patients, respectively, showed ≥50% reduction from baseline in palpable spleen length (Figure 1). The Kaplan–Meier estimated probabilities of maintaining ≥50% reduction from baseline in palpable spleen length were similar across DIPSS risk groups (Week 48, 83–92%; Week 72, 70–90%); 1.0% of low‐risk, 13.3% of Int‐1‐risk, 8.5% of Int‐2‐risk, and 1.7% of high‐risk patients had a spleen that became nonpalpable. Median time to first ≥50% reduction in spleen length in the overall population was 5.1 (range, 2.6–236.1) weeks and was longest in low‐ and high‐risk patients (low risk, 8.0 weeks; Int‐1 risk, 4.6 weeks; Int‐2 risk, 7.1 weeks; high risk, 8.1 weeks). Overall, 17.1% of patients had loss of response (return of spleen length to baseline level after ≥50% reduction) at any time during follow‐up, with similar rates among the risk groups.

Clinically meaningful symptom improvements were seen as early as 4 weeks after treatment initiation and were maintained throughout the study. In the FACT‐Lym TS, 17%–55% of patients achieved a response at each time point. On the FACIT‐Fatigue Scale, 43%–57% of patients achieved a response at each time point. A higher proportion of high‐risk patients achieved a response in both the FACT‐Lym TS and the FACIT‐Fatigue Scale versus low‐, Int‐1‐, and Int‐2‐risk patients. This was expected given that high‐risk patients had a higher symptom burden at baseline; however, symptom burden in high‐risk patients at Week 48 continued to be higher than in lower‐risk patients.

Overall, 191 deaths occurred during the study treatment period. The most common primary causes of death across the groups were MF (*n* = 35), pneumonia (*n* = 12), sepsis (*n* = 12), cardiac arrest (*n* = 11), and septic shock (*n* = 10). Median OS was longer for Int‐2‐risk patients than for high‐risk patients (253.6 vs. 147.3 weeks) but was not reached in low‐ or Int‐1‐risk patients. Estimated OS probability was 92% (95% confidence interval [CI]: 90–93) at Week 72 and 75% (95% CI: 70–80) at Week 240 (4.6 years). Rates of OS were lowest in high‐risk patients (19% at Week 240) compared with low‐risk (92%), Int‐1‐risk (89%), and Int‐2‐risk patients (61%). Estimated LFS probability was 90% (95% CI: 89–92) at Week 72 and 72% (95% CI: 67–77) at Week 240. Similarly, LFS rates were lowest in high‐risk patients (19% at Week 240) compared with low‐risk (92%), Int‐1‐risk (86%), and Int‐2‐risk patients (58%). In total, 37 patients developed acute myeloid leukemia during the study or within 28 days following study discontinuation, with an overall incidence rate of 0.7 per 100 patient‐treatment years. Estimated PFS probability was 87% (95% CI: 85–88; median follow‐up 57 weeks) at Week 72 and 66% (95% CI: 60–71) at Week 240. The survival rates at Week 240 were highest in low‐risk (90%) and Int‐1‐risk patients (82%) compared with Int‐2‐risk (46%) and high‐risk patients (15%).

Overall, 58.1% (*n* = 1071) of patients completed treatment and 31.7%, 34.9%, 46.2%, and 58.8% of low‐, Int‐1‐, Int‐2‐, and high‐risk patients, respectively, discontinued treatment. The primary reasons for treatment discontinuation included AEs, disease progression, death, and physician's decision (Table 2). Of the AEs leading to discontinuation in low‐risk patients, 10.0% had a suspected relationship to ruxolitinib (9.0% in Int‐1‐risk, 8.6% in Int‐2‐risk, and 12.9% in high‐risk patients). The most common hematologic AEs leading to discontinuation, regardless of relationship with ruxolitinib, were thrombocytopenia (low risk, 0%; Int‐1 risk, 2.4%; Int‐2 risk, 3.4%; and high risk, 6.2%) and anemia (low risk, 1.7%; Int‐1 risk, 1.4%; Int‐2 risk, 2.3%; and high risk, 3.1%). The most common nonhematologic AEs leading to discontinuation were infection (low risk, 3.3%; Int‐1 risk, 2.0%; Int‐2 risk, 2.6%; and high risk, 3.6%) and respiratory disorders (low risk, 0%; Int‐1 risk, 1.7%; Int‐2 risk, 1.3%; and high risk, 2.1%). Median drug exposures were 25.8, 16.3, 11.0, and 8.7 months, and the mean average daily doses were 30.4, 29.5, 28.1, and 28.8 mg in low‐, Int‐1‐, Int‐2‐, and high‐risk patients, respectively. Most patients started treatment at 20 mg b.i.d. (low risk, 66.7%; Int‐1 risk, 68.3%; Int‐2 risk, 57.0%; and high risk, 58.2%) or 15 mg b.i.d. (low risk, 26.7; Int‐1 risk, 25.4%; Int‐2 risk, 32.3%; and high risk 33.5%), and the majority had ≥1 dose reduction/interruption (low risk, 70.0%; Int‐1 risk, 69.1%; Int‐2 risk, 71.0%; and high risk, 72.7%).

**TABLE 2:** Reasons for treatment discontinuation

Reason for treatment discontinuation, *n* (%)	DIPSS risk category			
	Low (*n* = 60)	Int‐1 (*n* = 835)	Int‐2 (*n* = 755)	High (*n* = 194)
Any	19 (31.7)	291 (34.9)	349 (46.2)	114 (58.8)
AE	8 (13.3)	129 (15.4)	131 (17.4)	53 (27.3)
Disease progression	2 (3.3)	59 (7.1)	86 (11.4)	24 (12.4)
Death	1 (1.7)	20 (2.4)	41 (5.4)	25 (12.9)
Physician decision	3 (5.0)	27 (3.2)	46 (6.1)	3 (1.5)
Other^a^	5 (8.3)	56 (6.7)	45 (6.0)	9 (4.6)

*Note:* Percentages are given as percentage of the total number of patients in each group.

Abbreviations: AE, adverse event; DIPSS, Dynamic International Prognostic Scoring System; Int, intermediate.

^a^ Other included withdrawal of consent, loss to follow‐up, administrative problems, and protocol deviation.

**FIGURE 2:**
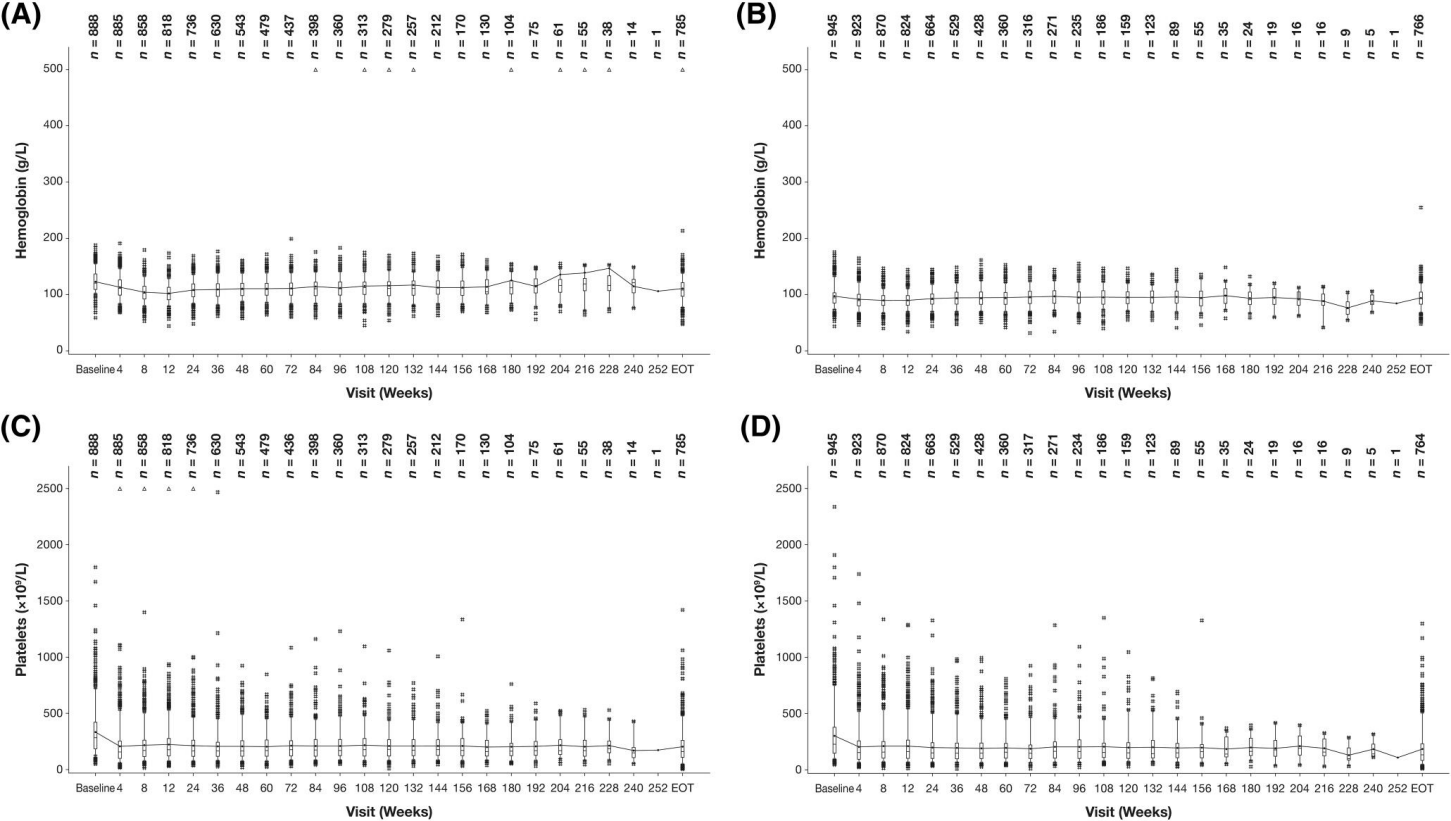
Hemoglobin levels and platelet counts over time by DIPSS risk group. Hemoglobin by (A) low + Int‐1 risk and (B) Int‐2 + high risk and platelet count by (C) low + Int‐1 risk and (D) Int‐2 + high risk. DIPSS, Dynamic International Prognostic Scoring System, Int, intermediate

The most common hematologic AEs across all DIPSS categories were anemia and thrombocytopenia, with the highest rates of Grade ≥3 anemia and thrombocytopenia seen in high‐risk patients (Table 3). Across the DIPSS risk groups, median hemoglobin levels decreased from baseline (106 g/L) to a nadir from Weeks 4 to 24 (94–99 g/L across the four time points), but they increased to near‐baseline levels after Week 36 (Figure 2A,B). Median platelet counts decreased from baseline (257 × 10^9^/L) during the first 4 weeks, with a nadir of 154 × 10^9^/L, and remained stable over time (Figure 2C,D). Serious AEs were reported by 28.3%, 30.4%, 40.7%, and 60.8% of the low‐, Int‐1‐, Int‐2‐, and high‐risk patients, respectively. The most common hematologic AEs across all risk groups were Grade ≥3, with high‐risk patients reporting significantly greater rates of all Grade ≥3 AEs (Table 3).

**TABLE 3:** Exposure‐adjusted AEs regardless of study drug relationship (in ≥2% of patients)^a^

Preferred term	DIPSS risk category				
	Low	Int‐1	Int‐2	High	
	(*n* = 60)	(*n* = 835)	(*n* = 755)	(*n* = 194)	
	Grade ≥3 IR^b^	Grade ≥3 IR^b^	Grade ≥3 IR^b^	Grade ≥3 IR^b^	
Any AE	31.4	48.5	116.7	244.1	
Hematologic AEs					
Anemia	10.4	14.9	53.3	92.8	
Thrombocytopenia	3.2	6.9	14.9	24.6	
Neutropenia	2.4	2.2	3.8	7.1	
Hemoglobin decreased	1.6	0.7	0.7	6.9	
Leukocytosis	0	0.7	1.0	6.3	
Platelet count decreased	0.8	1.5	1.7	3.2	
Leukopenia	0.8	1.3	2.6	2.3	
Nonhematologic AEs					
Pneumonia	0	2.3	4.0	8.7	
Dyspnea	0	1.0	1.3	5.9	
Cardiac failure	1.6	0.9	1.2	4.5	
Acute kidney injury	0	0.3	0.7	3.6	
Abdominal pain	0	0.3	1.2	3.2	
Sepsis	0	0.7	1.3	3.1	
Pyrexia	0	0.7	2.7	2.7	
Cardiac arrest	0.8	0.1	0.6	2.7	
Asthenia	2.4	1.0	1.8	2.3	
Urinary tract infection	0	0.5	1.1	2.3	

Abbreviations: AE, adverse event; Int, intermediate: IR, incidence rate.

^a^ IR (exposure‐adjusted incidence rate per 100 patient‐years): number of patients with an event divided by the corresponding sum of the exposure duration for all patients, where duration of exposure in patient‐treatment years is counted up to the first qualifying event (or end of time at risk for subjects without event [i.e., last treatment plus safety follow‐up period]).

^b^ AEs occurring ≤28 days of treatment discontinuation are included.

## 4 DISCUSSION

The JUMP study included the largest cohort of patients with MF treated with ruxolitinib to date, including patients with low‐ and Int‐1‐risk MF who were not included in the Phase 3 COMFORT studies.^2^, ^3^ This analysis demonstrated that ruxolitinib was generally well tolerated by patients across all DIPSS risk groups and had an AE profile consistent with previous reports.^2^, ^3^, ^4^ Most patients also experienced reductions in splenomegaly and symptoms, irrespective of risk group.

In addition to the previously demonstrated benefit of ruxolitinib in higher risk patients in the COMFORT I and II studies, this analysis demonstrates that ruxolitinib also confers clinical benefit in lower risk patients. In this analysis, time to first ≥50% reduction in spleen length was independent of DIPSS risk status and was longest in low‐ and high‐risk patients. A slightly greater proportion of lower‐risk patients achieved larger spleen size reductions and had the shortest median time to first spleen response, which may correlate with better OS rates.^14^ These results are comparable to previously reported studies suggesting that patients with less‐advanced MF who initiate ruxolitinib therapy have improved outcomes and that patients with higher‐risk disease have lower spleen response rates.^15^, ^16^ Results from other smaller‐scale studies also indicate a benefit of ruxolitinib in lower‐risk (Int‐1 risk) patients.^17^ In ROBUST, an open‐label Phase 2 study that included 14 patients with Int‐1 MF, similar findings were observed, with 50% of Int‐1‐risk patients achieving ≥50% reduction in palpable spleen length by Week 48. At the same time point, 21.4% of these patients had ≥50% decrease in MF Symptom Assessment Form total symptom score, although this finding was confounded by the low number of patients with data available at Week 48.^18^ An additional retrospective, real‐world observational review of US medical records included 108 patients with low/Int‐1 risk based on IPSS. Most of the Int‐1‐risk patients included in the study experienced reduction in symptom severity, and the percentage of patients with palpable spleen ≥10 cm reduced from 51% at baseline to 10% at best response. Rates of anemia and thrombocytopenia were similar to those observed in the current analysis.^19^ Similarly, a report of 70 Int‐1‐risk patients treated with ruxolitinib, according to standard clinical practice, showed rates of spleen and symptom response of 55% and 80%, respectively; however, rates of Grade 3 anemia were higher and rates of thrombocytopenia were lower than those observed in the current analysis (40.6% and 2.9%, respectively).^20^ Finally, a Phase 2 study assessing alternative dosing regimens of ruxolitinib that included a large percentage of Int‐1‐risk patients (69%) also demonstrated efficacy in reduced spleen volume and symptom score, with a similar safety profile.^21^

A higher proportion of patients in the low‐risk group (73.3%) had platelet counts ≥200 × 10^9^/L than in the high‐risk group (57.2%), and a majority of high‐risk patients (56.7%) had received prior transfusions. However, despite their poorer physical condition, a high proportion of high‐risk patients achieved a symptom response in the current analysis.

Lower risk patients experienced improvements in symptoms and fewer discontinued treatment due to AEs. These observations suggest that early intervention in patients with MF may lead to lower symptom burden and better quality of life.^22^, ^23^ In a real‐world study in Italy, significantly higher response rates were observed in Int‐1‐risk patients compared with Int‐2‐ or high‐risk patients, with significantly lower progression and death rates. The authors concluded that the improved responses in these patients may be due to earlier use of ruxolitinib in the disease course and welcomed further prospective studies investigating this finding.^24^ Evidence from the current analysis also suggests that early use of ruxolitinib could provide benefits in lower‐risk patients, because both symptom and spleen responses were observed in this risk group. These findings, together with observations from previous studies on survival benefits and impacts on disease course in some individuals,^17^ indicate that early initiation of therapy in patients with intermediate‐ or high‐risk disease has the potential to maximize long‐term therapy benefits associated with ruxolitinib.

## 5 CONCLUSION

Overall, the findings from this analysis suggest that ruxolitinib is well tolerated in DIPSS lower risk patients with MF and may be of benefit to this population in addition to the previously demonstrated benefits in higher risk patients.

## CONFLICT OF INTERESTS

Francesco Passamonti has received research funding from and served on speaker bureaus for Celgene and Novartis. Vikas Gupta has received consultancy fees, honoraria, and research funding from Novartis and has received consultancy fees and research funding from Incyte. Lynda Foltz has received consultancy fees from Pfizer; research funding from Gilead, Incyte, and Promedior; and consultancy fees, honoraria, and research funding from Celgene and Novartis. Andrey Zaritskey has received consultancy fees from Janssen and Novartis and has served on speaker bureaus for Novartis. Haifa Kathrin Al‐Ali has received consultancy fees, honoraria, and research funding from Celgene and Novartis; honoraria from Alexion; and consultancy fees and honoraria from Gilead. Renato Tavares has received consultancy fees from Novartis. Pia Raanani has received grants from Ariad (Medison), Novartis, and Pfizer and has received consultancy fees from and has served on advisory boards for Ariad (Medison), Bristol‐Myers Squibb, Novartis, and Pfizer. Martin Griesshammer has received consultancy fees and honoraria from and has served on speaker bureaus for AOP Orphan, Baxalta, Gilead, Novartis, and Shire and has received honoraria from and has served on speaker bureaus for Sanofi. Catherine Bouard, Carole Paley, and Ranja Tiwari are employees of Novartis. Alessandro M. Vannuchi has served on speaker bureaus for Gilead and Shire and has served on the board of directors or advisory committees for, has received research funding from, and has served on speaker bureaus for Novartis. The other authors declare that there are no conflict of interests.

## ETHICS STATEMENT

The study was approved by the institutional review board at each participating institution and conducted in accordance with applicable local regulations and the principles of the Declaration of Helsinki. All patients provided written informed consent before entry into the study.

## AUTHOR CONTRIBUTIONS

Francesco Passamonti, Vikas Gupta, Bruno Martino, Lynda Foltz, Andrey Zaritskey, Haifa Kathrin Al‐Ali, Renato Tavares, Margherita Maffioli, Pia Raanani, Pilar Giraldo, Martin Griesshammer, Paola Guglielmelli, and Alessandro M. Vannucchi enrolled patients, performed research, and contributed to data collection, analysis, and interpretation. Catherine Bouard and Carole Paley contributed to data interpretation. Ranjan Tiwari performed statistical analyses and contributed to data interpretation. All authors critically reviewed each version of the manuscript and approved the final version for submission.

### PEER REVIEW

The peer review history for this article is available at https://publons.com/publon/10.1002/hon.2898.

## Data Availability

Data included in this manuscript were previously presented at the European Hematology Association Congress in 2016, 2017, and 2018. Novartis is committed to sharing with qualified external researchers access to patient‐level data and supporting clinical documents from eligible studies. These requests are reviewed and approved by an independent review panel on the basis of scientific merit. All data provided are anonymized to respect the privacy of patients who have participated in the trial, in line with applicable laws and regulations. This trial data availability is according to the criteria and process described on www.clinicalstudydatarequest.com.
